# Supplementary material for: Complex‐centric proteome profiling by SEC‐SWATH‐MS
Source: Mol Syst Biol. 2019 Jan 14;15(1):e8438. doi: 10.15252/msb.20188438 (PMC6346213; doi:10.15252/msb.20188438)
Supplement: Supplementary file 8 — Dataset EV7 [file MSB-15-e8438-s008.zip › feature_plots_string/Q15583.pdf]

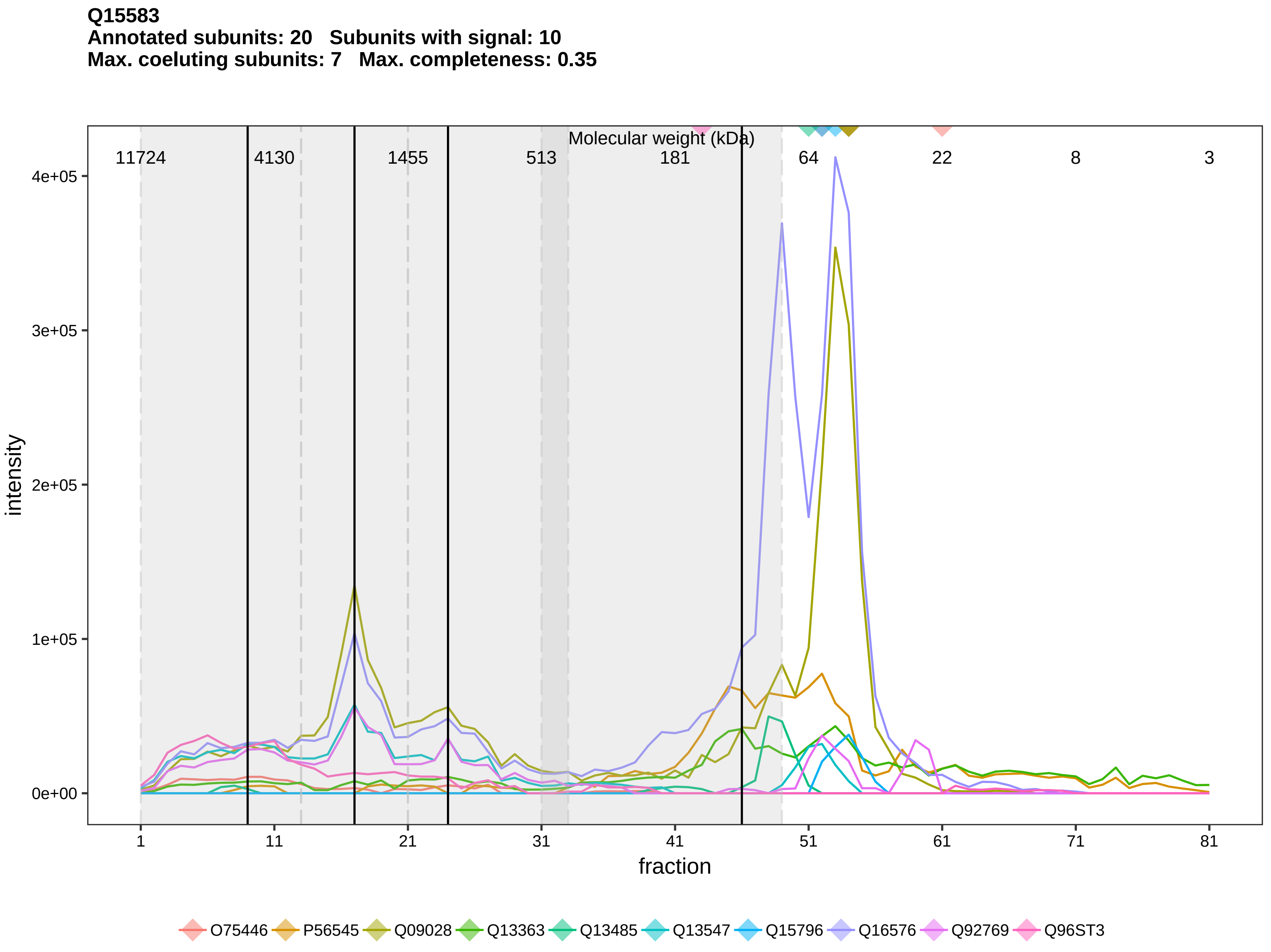
Q15583
Annotated subunits: 20 Subunits with signal: 10
Max. coeluting subunits: 7 Max. completeness: 0.35
Molecular weight (kDa)
11724
4130
1455
513
181
64
22
8
3
intensity
4e+05
3e+05
2e+05
1e+05
0e+00
fraction
1
11
21
31
41
51
61
71
81
O75446
P56545
Q09028
Q13363
Q13485
Q13547
Q15796
Q16576
Q92769
Q96ST3
